# Re-sensitizing Multidrug Resistant Bacteria to Antibiotics by Targeting Bacterial Response Regulators: Characterization and Comparison of Interactions between 2-Aminoimidazoles and the Response Regulators BfmR from *Acinetobacter baumannii* and QseB from *Francisella* spp.

**DOI:** 10.3389/fmolb.2018.00015

**Published:** 2018-02-13

**Authors:** Morgan E. Milton, Bradley M. Minrovic, Danni L. Harris, Brian Kang, David Jung, Caleb P. Lewis, Richele J. Thompson, Roberta J. Melander, Daina Zeng, Christian Melander, John Cavanagh

**Affiliations:** ^1^Discovery Sciences, RTI International, NC, United States; ^2^Department of Chemistry, North Carolina State University, Raleigh, NC, United States; ^3^Agile Sciences, Inc., Raleigh, NC, United States; ^4^Department of Molecular and Structural Biochemistry, North Carolina State University, Raleigh, NC, United States

**Keywords:** biofilms, antibiotic resistance, two-component systems, response regulators, *Acinetobacter baumannii*, Francisella

## Abstract

2-aminoimidazole (2-AI) compounds inhibit the formation of bacterial biofilms, disperse preformed biofilms, and re-sensitize multidrug resistant bacteria to antibiotics. 2-AIs have previously been shown to interact with bacterial response regulators, but the mechanism of interaction is still unknown. Response regulators are one part of two-component systems (TCS). TCSs allow cells to respond to changes in their environment, and are used to trigger quorum sensing, virulence factors, and antibiotic resistance. Drugs that target the TCS signaling process can inhibit pathogenic behavior, making this a potent new therapeutic approach that has not yet been fully exploited. We previously laid the groundwork for the interaction of the *Acinetobacter baumannii* response regulator BfmR with an early 2-AI derivative. Here, we further investigate the response regulator/2-AI interaction and look at a wider library of 2-AI compounds. By combining molecular modeling with biochemical and cellular studies, we expand on a potential mechanism for interaction between response regulators and 2-AIs. We also establish that *Francisella tularensis/novicida*, encoding for only three known response regulators, can be a model system to study the interaction between 2-AIs and response regulators. We show that knowledge gained from studying *Francisella* can be applied to the more complex *A. baumannii* system, which contains over 50 response regulators. Understanding the impact of 2-AIs on response regulators and their mechanism of interaction will lead to the development of more potent compounds that will serve as adjuvant therapies to broad-range antibiotics.

## Introduction

The formation of biofilms contributes to significant bacterial persistence in the environment, pathogenicity, and resistance to antimicrobials (Donlan, 2002). Bacteria spend an estimated 80% of their time in a biofilm state, adhering to surfaces, and one another. A biofilm is composed of an extracellular matrix which provides protection against a variety of physical and chemical assaults. In a biofilm state, bacteria can be up to 1,000-fold more resistant to antibiotics than their planktonic counterparts (Donlan and Costerton, 2002; Rasmussen and Givskov, 2006; Percival et al., 2011). Understanding how biofilms are formed and developing small molecule therapies are vital steps in combating antibiotic resistance.

*Acinetobacter baumannii* and *Francisella* species are of particular interest in studying the impact of biofilms on human health. *A. baumannii* is highly prevalent in hospitals and has shown extensive multi-drug resistance in the clinical setting (Dijkshoorn et al., 2007; Perez et al., 2007). *A. baumannii* belongs to a group of pathogens known as the ESKAPE pathogens, named as such because the bacteria easily “escape” antibiotics through the rapid acquisition of resistance (Rice, 2008). Recently, the World Health Organization has listed *A. baumannii* as a critical priority for combating antibiotic resistant bacteria (World Health Organization, 2017). On the other hand, while infection by *Francisella* species is less common, *Francisella tularensis/tularensis* is listed by the Centers for Disease Control and Prevention as a Category A select agent (Sjöstedt, 2007). Its ease of aerosolization, high infectivity, and ability to quickly incapacitate those infected makes *F. tularensis/tularensis* a highly viable biowarfare agent. Both bacteria utilize biofilms to increase their persistence, pathogenicity, and antibiotic resistance (Durham-Colleran et al., 2010; Imperi et al., 2011; McConnell et al., 2013; Sutera et al., 2014; Kröger et al., 2016).

The response regulator proteins BfmR and QseB are responsible for controlling biofilm formation as well as degrees of antibiotic resistance in *A. baumannii* and *F. tularensis/novicida* (the mouse model of *F. tularensis/tularensis*), respectively. *Francisella* species encode an exceptionally small number of response regulators (Larsson et al., 2005) compared to other bacteria. This reduced complexity makes *F. tularensis/novicida* an excellent system to the cellular effects of targeting response regulators with small molecule therapies. Response regulators work in combination with a sensor kinase to form the ubiquitous communication two component system (TCS) scheme (Stock et al., 2000). Typically, the sensor kinase is a transmembrane histidine kinase that detects an external signal. This response triggers an autophosphorylation event. The phosphate group is subsequently transferred to a partner response regulator, changing it from an “inactive” to “active” state. The activated response regulator propagates the signal through transcriptional regulation. Response regulators are phosphorylated at a conserved site in the N-terminal receiver domain. A variable C-terminal DNA-binding domain facilitates binding to target DNA promotor sites. A highly flexible linker region of varying length connects these two domains. It is common for response regulators to be monomers in solution until activation triggers dimerization of the receiver domain. This brings the two DNA-binding domains in closer proximity to better bind the two half sites of the cognate promoter (Gao and Stock, 2009).

Derivatives of a cell-permeable, non-toxic family of 2-AIs are known to interact with response regulators (Thompson et al., 2012; Stowe et al., 2015; Milton et al., 2017). This class of compounds has been widely shown to inhibit and disperse biofilms, and also to work as an adjuvant therapy with traditional antibiotics to re-sensitize multidrug-resistant bacteria (Ballard et al., 2008; Richards et al., 2008a,b; Rogers and Melander, 2008; Brackett et al., 2014). Adjuvants act as a complementary therapy to antibiotic treatment. Their use has been proposed to extend the lifespan of antibiotics and reduce further resistance. The potential to re-sensitize bacteria to antibiotics makes adjuvants a powerful tool against the ever increasing antibiotic resistance (Wright, 2016; González-Bello, 2017; Melander and Melander, 2017). Understanding how potential adjuvant compounds function within the cell will aid in the development of more potent therapies. The specific mechanism through which 2-AIs interact with response regulators is still relatively unknown.

We first identified that an early 2-AI derivative could interact with the N-terminal and C-terminal domains of BfmR, as well as with full length protein (Thompson et al., 2012). We probed the interactions between response regulators and 2-AIs by validating QseB as a cellular target for the compounds (Milton et al., 2017). This provided the first direct evidence that QseB was binding to 2-AIs, and that 2-AIs impacted QseB-specific cellular functions, biofilm formation and β-lactam resistance.

Here we provide further evidence that BfmR is a cellular target of 2-AIs. Based on our previous findings with QseB, we propose that *F. tularensis/novicida* can act as a model organism for studying how 2-AIs interact with response regulators within the more complicated *A. baumannii* system. Additionally, understanding the differences between the two systems will aid in the development of organism specific and broad range adjuvant therapies. Here, we combine cellular, biochemical, and molecular dynamics techniques to further elucidate the mechanism of action of 2-AIs and response regulators. These findings will aid in the development of more potent compounds that can act as broad range or specific adjuvant therapies.

## Materials and methods

### Bacterial strains, media, and antibiotics

*A. baumannii* 19606 and 1605 were obtained from ATCC as 19606 and BAA-1605, respectively. *A. baumannii* strain 5075 was obtained from the Manoil lab at the University of Washington. Cells were grown in LB at 37°C for biofilm assays and Muller Hinton Broth 2 for MIC assays. Antibiotics were purchased from Sigma-Aldrich.

### Cloning, expression, and purification

The coding region of *bfmR* from *A. baumannii* strain 19606 was in the expression vector pET28a (Novagen). Protein was over-expressed in BL21(DE3)pLysS cells at 37°C in LB. At an OD_600_ of 0.6-0.8, cells were induced with 1 mM isopropyl β-D-thiogalactopyranoside (IPTG) at 30°C, for 4 h. Harvested cell pellets were stored at −80°C for later use.

BfmR pellets were resuspended in lysis buffer (20 mM Tris pH 7.9, 400 mM NaCl, and 5 mM imidazole) at 10 mL g^−1^ of pellet. Cells were sonicated and the resulting lysate clarified at 20,400 × g for 15 min. Clarified lysate was loaded onto 10 mL of Ni-NTA resin (QIAGEN) pre-equilibrated in lysis buffer. Bound protein was washed with 10 column volumes of lysis buffer and 10 column volumes of 20 mM Tris pH 7.9, 1 M NaCl, and 15 mM imidazole. The protein was eluted with a linear gradient from lysis buffer to elution buffer (20 mM Tris pH 7.9, 400 mM NaCl, and 300 mM imidazole). Fractions containing protein were pooled and dialyzed into 20 mM Tris pH 7.9 and 200 mM NaCl. The affinity tag was cleaved by 100 units of thrombin for 2 h at room temperature. Cleavage was quenched with 0.1 mM AEBSF and sample continued in dialysis of 20 mM Tris pH 7.9 and 200 mM NaCl.

### Thermal shift assays (TSA)

The compounds were dissolved in 100% PEG 400 to a final concentration of 1 mM. Reactions were carried out using final concentrations of 5 μM BfmR, 25 μM compound, 10% v/v PEG 400 and 10x SYPRO orange (ThermoFisher Scientific). Samples were prepared in three technical replicates on a CFX384 Touch Real-Time PCR Detection System (BioRad). Samples were heated from 25 to 95°C in 0.5°C increments, holding for 30 s at each step. Fluorescence was detected using the default HEX wavelengths. Data was fit to a Boltzmann curve using SigmaPlot. Assays were repeated in triplicate.

### Biofilm inhibition assays

Overnight cultures of *A. baumannii* 19606 in LB were subcultured to an OD_600_ of 0.01 in the same media. Compounds were added from stock solutions to give the desired concentrations to be tested. Inoculum with no compound added served as the untreated control. Samples were aliquoted (100 μL) into the wells of the 96-well PVC microtiter plate. Sample plates were then wrapped in plastic and incubated under stationary conditions for 24 h at 37°C. After incubation, the plates were visually inspected for the presence of consistent bacterial growth. The medium was discarded from the wells and the plates were washed thoroughly with water. Plates were then stained with 110 μL of 0.1% aqueous crystal solution violet (CV) and incubated at ambient temperature for 30 min. Plates were washed with water again. The remaining stain was solubilized with 200 μL of 100% ethanol and incubated again at ambient temperature for 10 min. A sample of 125 μL of solubilized CV stain from each well was transferred to the corresponding wells of a polystyrene microtiter dish. The absorbance of each well was measured at 540 nm and biofilm inhibition was quantified by calculating the 540 nm absorbance of treated wells as a percentage of untreated control wells. The IC_50_ value was defined as the concentration of compound at which a 50% reduction in biofilm formation was observed compared to the untreated control. Assays were repeated with between three and eight biological replicates.

### Minimum inhibitory concentration (MIC) assays

MIC assays were performed according to Clinical and Laboratory Standards Institute (CLSI) standards. MIC values of the compounds alone were established prior to studies with antibiotics *A. baumannii* cells were pre-incubated with 2-AIs for 30 min prior to assessment in the MIC assay. Assays were initially repeated with two biological replicates. Compounds that showed MIC lowering activity were further repeated.

### Docking and MMGBSA rescoring

Both Autodock Vina (Trott and Olson, 2010) and Schrodinger GLIDE-XP (Friesner et al., 2006) docking approaches were employed in a large region encompassing the N-and C-terminal domains of QseB, PmrA, and BfmR. When employing Autodock Vina, AMBER16 (Case et al., 2016) models of the systems were prepared using TLEAP and the systems energy minimized for 800 steps employing steepest descents followed by 8,000 steps of conjugate gradient minimization using the ff14SB forcefield. In the case of Autodock Vina, potential binding sites were first detected using blind docking. Here we employed large docking region (ca 30 × 30 × 30 Å^3^), completely encompassing the N/C domains. Inhibitor binding pose regions located using this approach were then subsequently re-investigated using smaller docking boxes for finer sampling. In all cases, a Autodock Vina high exhaustiveness setting of 80 was used to sample inhibitor/response-regulator configurations and a total of 20 top-Vina score docking poses saved for subsequent molecular mechanics generalized Born surface area (MMGBSA) rescoring described below. In house test problems, including PDB Bind high quality crystal structures, reveal that this hybrid Vina+MMGBSA approach recovers the crystallographically relevant poses (configurations) as the lowest MMGBSA scored docking pose ca. 75% of the time (with RMSD <2 Å). Additionally, the lowest MMGBSA score has improved affinity correlation compared to docking scores (Hou et al., 2011; Greenidge et al., 2013; Zhang et al., 2014). Parallel exploration using Schrodinger GLIDE-XP first identified potential binding sites in response regulator models using SiteMap on systems prepared with ProteinPrep energy minimized with 2,500 steps of conjugate gradients and collection of 5–10 top GLIDE-XP scored poses. Predicted inhibitor binding locations for these two (Vina+MMGBSA/GLIDE-XP) distinct approaches were found to be in agreement with small differences in specific residue interaction motifs beyond the scope of the present discussion.

### MMGBSA-min and MMGBA-SA

Docking poses from Autodock Vina or GLIDE-XP are subjected to MMGBSA rescoring employing a combination of AMBER16 (Case et al., 2016) driven by our own C++ and bash script base. Our automated workflow: (1) collected docking poses of all ligands (in mol2 format), (2) performed ligand formal charge perception (C++/OpenBabel), (3) quantum chemical determination of each of the ligand charges using AM1-BCC, (4) determination of additional internal ligand force field parameters using AMBER-GAFF, (5) energy minimized poses in complexes with the receptor/protein employing a GPU/CPU hybrid MD-simulated annealing (300K MD followed by energy minimization) procedure before computing the MMGBSA/MMPBSA score.

### Molecular dynamics of response regulator systems

AMBER16 (Case et al., 2016) was used to prepare the coordinates of PmrA. Coordinates were extracted from PDB ID 4S05 with the DNA removed. The initial system, prepared using the TLEAP module along with the ff14SB forcefield, was immersed in boxes of TIPS3P waters. A distance of 15 Å around any protein heavy atom was used as the criterion of choosing the box size. The system was energy minimized for 8,000 steps of conjugate gradient minimization, and then heated to 300K over 200 ps using the NPT ensemble, while harmonically constraining the protein atoms to initially equilibrate the water box density. After energy minimization, the system was allowed 5 ns of unconstrained equilibration at constant volume and temperature (300K). After equilibration, the PmrA system was employed in solvated dynamics in aqueous solution. Dynamics was explored at 300K for 250 ns. The structures were saved every 5 ps. The structural evolution in solution of the initial “extended” conformation in the absence of DNA was examined both in principal component analysis of the dynamics coordinate as well as clustering the backbone coordinates of PmrA over the 250 ns timeframe using the average-linker algorithm to obtain approximate populations of the top 5-clusters as well as representative PDB-snapshots for the clustered families.

## Results and discussion

### 2-AIs bind QseB and BfmR

**Figure 1:**
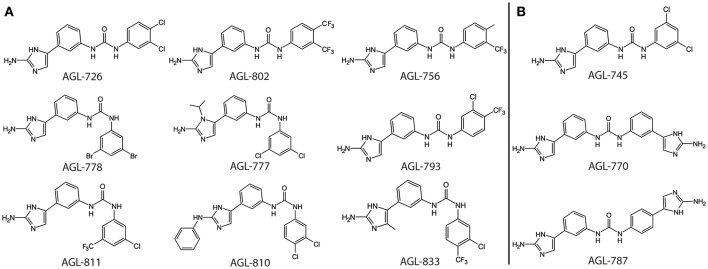
Top binding compounds for QseB and BfmR based on protein thermal shift. **(A)** Compounds that significantly increased the T_m_ of both QseB and BfmR. Compounds are grouped based on structural similarities. **(B)** Additional compounds that interact with BfmR. 753 and 782 are not shown due to patent pending.

The formation of a protein-ligand complex is often associated with an increase in protein stability. Complex formation can be determined by measuring the change in the protein's melting temperature (T_m_), a strategy generally used in drug discovery (Pantoliano et al., 2001; Lo et al., 2004; Niesen et al., 2007). Previously, we developed a high-throughput fluorescence based thermal shift assay to evaluate the binding of 2-AIs to QseB (Milton et al., 2017). This same assay was performed with BfmR to identify binding interactions (Supplementary Figures 1, 2). As a result, compounds that bind QseB tightly enough to induce a significant change in the T_m_ also bind BfmR (Figure 1). AGL-726, 778, 793, 802, 753, 777, 811, 810, 756, 833, and 782 increase the T_m_ of QseB and BfmR above background. Additionally, AGL-745, 770, and 787 showed binding potential for BfmR. This suggests that compounds may be designed to target a broad range of response regulators or be modified to interact with only a specific response regulator.

We previously observed with QseB that a compound can interact with a response regulator, but the ΔT_m_ upon binding does not change significantly above background levels (Milton et al., 2017). As a result, it is likely that a compound with a moderate to high binding affinity can be confidently detected using the thermal shift assay. This allows for quick identification of leads from a library of compounds.

### Biofilm inhibition

QseB and BfmR have been shown to play a central role in the regulation of biofilm formation in *F. tularensis/novicida* and *A. baumannii*, respectively (Tomaras et al., 2008; Durham-Colleran et al., 2010; Liou et al., 2014). Since *Francisella* species only encode three known response regulators, *F. tularensis/novicida* has the potential to be an excellent model system to study the effects of 2-AIs on response regulators. We previously demonstrated that AGL-600 and 726 inhibited the formation of *F. tularensis/novicida* biofilms (Milton et al., 2017). Due to the phenotypes associated with QseB knockouts (Durham-Colleran et al., 2010) and our direct evidence that these compounds bound QseB, we concluded that QseB was a target of the 2-AIs *in vitro*. As with QseB, deletion of *bfmR* results in a complete loss of biofilm formation (Tomaras et al., 2008). Since BfmR also directly binds the 2-AIs, we expected the compounds to have similar biofilm inhibition properties in *A. baumannii*.

**Figure 2:**
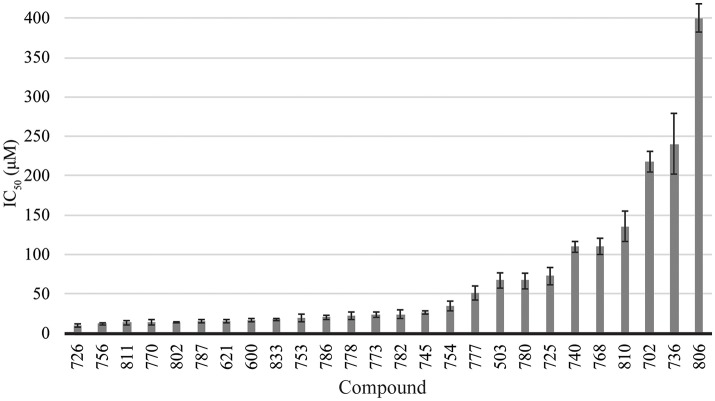
Biofilm inhibition by 2-AI compound library. Biofilm inhibition IC_50_ rankings for *A. baumannii* 19606 biofilms.

The library of compounds were screened for their ability to inhibit the formation of *A. baumannii* biofilms using the traditional crystal violet assay (O'Toole, 2011). All compounds inhibited biofilm formation to varying degrees (Figure 2). A majority of the compounds identified as BfmR binding partners ranked among the most potent inhibitors, with initial screening IC_50_ values for biofilm inhibition between 10 and 50 μM. This suggests a correlation between compounds that bind BfmR and compounds that reduce *A. baumannii* biofilm growth.

**Figure 3:**
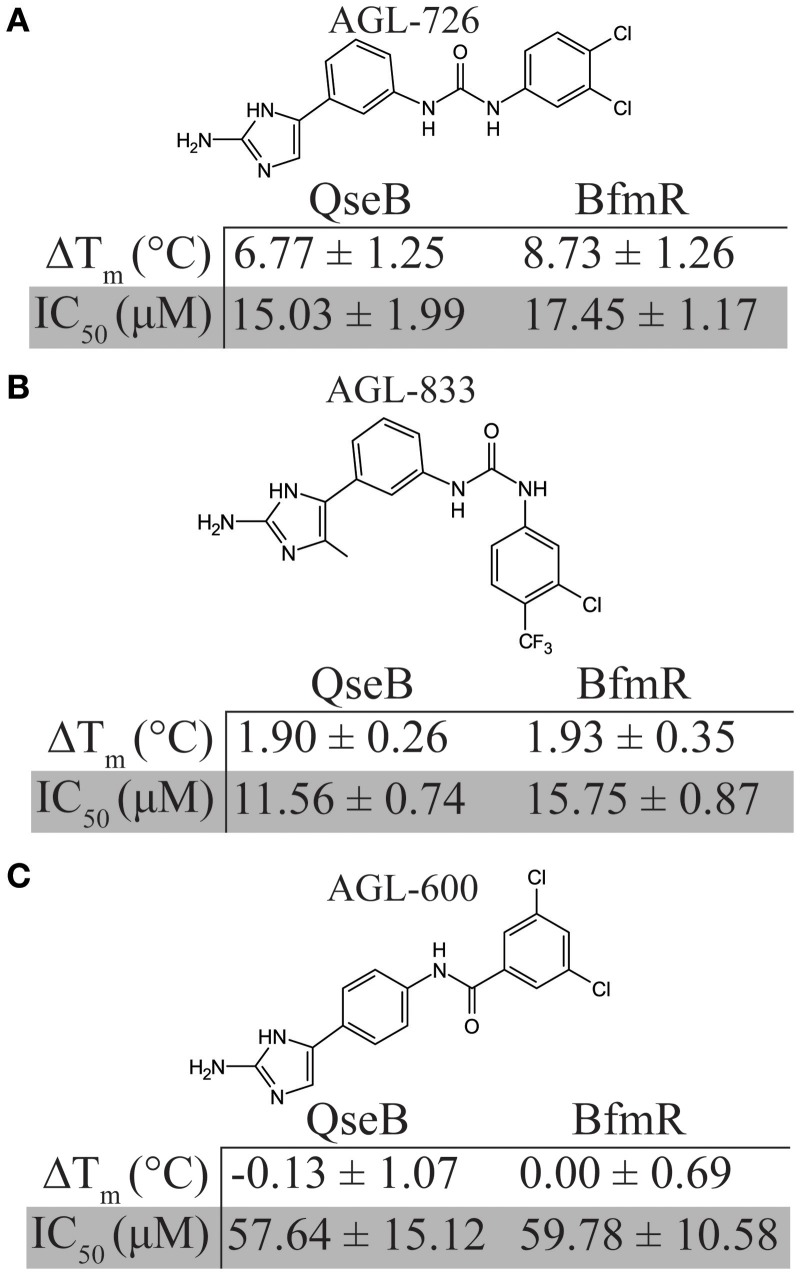
Comparison of biofilm inhibition and BfmR binding properties for three select compounds across bacterial species. **(A)** AGL-726 binds BfmR and QseB with the highest change in T_m_. This binding correlates with low IC_50_ values. **(B)** AGL-833 stimulates a minor increase in the T_m_s of BfmR and QseB while still having potent biofilm inhibition properties. **(C)** AGL-600 binds very weakly, if at all, to BfmR and QseB, which probably contributes to a higher IC_50_ values for biofilm inhibition.

Based on thermal shift data, three compounds were selected for further comparison between *A. baumannii* and *F. tularensis/novicida* biofilm inhibition. AGL-726 significantly increased the T_m_ values of QseB (Milton et al., 2017) and BfmR (Supplementary Figure 1) above background, with ΔT_m_ of 6.77 ± 1.25 and 8.73 ± 1.26°C, respectively. The compound is one of the most potent biofilm inhibitors with an IC_50_ of 15.03 ± 1.99 μM for *F. tularensis/novicida* and 17.45 ± 1.17 μM for *A. baumannii* (Figure 3A). To test a less extreme example, we investigated AGL-833. AGL-833 has a nearly identical ΔT_m_ for QseB and BfmR, 1.90 ± 0.26 and 1.93 ± 0.35°C, respectively. AGL-833 also proved to be a potent biofilm inhibitor with IC_50_ values of 11.56 ± 0.74 and 15.75 ± 0.87 μM for *F. tularensis/novicida* and *A. baumannii*, respectively (Figure 3B). Finally, AGL-600 was investigated. We previously reported that while AGL-600 binds QseB, binding manifested as an insignificant ΔT_m_ (Milton et al., 2017). Likewise, AGL-600 has little impact on the ΔT_m_ of BfmR (Supplementary Figure 1). The lower binding affinity correlates with a decrease in biofilm inhibition. AGL-600 inhibits *F. tularensis/novicida* with an IC_50_ of 57.64 ± 15.12 μM and *A. baumannii* with an IC_50_ of 59.78 ± 10.58 μM (Figure 3C). Overall, these results demonstrate that even minor modifications to the variable region of the compound can have significant impacts on the 2-AI's ability to bind response regulators and provide support and that, like QseB, BfmR is also a cellular target of 2-AIs.

### 2-AIs impact the minimum inhibitory concentration of antibiotics

Response regulators are often involved in antimicrobial resistance. For instance, QseB has been shown to play a role in polymyxin B resistance (Mohapatra et al., 2007). The addition of AGL-600 and AGL-726 were able to lower the minimum inhibition concentration (MIC) of polymyxin B in *F. tularensis/novicida* (Milton et al., 2017). This finding provided further evidence that QseB is a cellular target of 2-AIs.

BfmR has been shown to mediate resistance to meropenem and colistin (Russo et al., 2016). 2-AIs that interact with BfmR *in vivo* may impact antibiotic resistance. To test the effects of the library of 2-AIs on antibiotic resistance, MIC lowering activity was evaluated for two strains of *A. baumannii*. Strain 1605 is a multidrug resistant *A. baumannii* isolated from military casualties (Tien et al., 2007) and strain 5075 is a highly virulent isolate often used as a model strain to evaluate antimicrobial treatments (Jacobs et al., 2014). Both strains were tested for increased sensitivity to meropenem, imipenem, and doripenem in the presence of our library of 2-AIs (Supplementary Table 1). Many of the compounds that interact with BfmR in the thermal shift experiment have MIC lowering activity, specifically 726, 756, 770, 778, 786, 802, and 833. Five additional compounds which have biofilm inhibition IC_50_ values less than 100 μM (503, 600, 621, 754, and 773), also showed MIC lowering activity. These results further support that the response regulator BfmR is a cellular target of 2-AIs, and that the compounds can act as adjuvant therapies.

AGL-600, 726, and 833 all had potent MIC lowering activity (Table 1). All three compounds reduce the MIC in a dose-dependent manner. AGL-833 had to be used at lower concentrations due to having a lower MIC value than AGL-600 and 726 when tested in the absence of antibiotics, 12.5, 50, and 50 μM, respectively. Regardless, AGL-833 proved to be a potent MIC lowering adjuvant. Deletion of *bfmR* results in roughly a 2-fold lower MIC for meropenem than WT *A. baumannii* strain AB307-0294 (Russo et al., 2016). At concentrations four times lower than the MIC values of the 2-AIs alone, these compounds were able to reduce the MIC values of three carbapenem antibiotics at or beyond what was observed in a *bfmR* deletion mutant. While resistance likely varies greatly between strains, it is highly probable that the MIC lowering activity seen in these compounds can be attributed to BfmR being a cellular target.

Table 1MIC lowering activity of AGL-600, 726, and 833.

**Table:** 

		**AGL-600**					
		***A. baumannii*** **1605**			***A. baumannii*** **5075**		
**Antibiotics**		**0 μM**	**30 μM**	**60 μM**	**0 μM**	**30 μM**	**60 μM**
Imipenem	MIC (μg/mL)	32	–	4	32	2	–
	Fold reduction			**8**		**16**	
Meropenem	MIC (μg/mL)	32	–	4	32	2	–
	Fold reduction			**8**		**16**	
Doripenem	MIC (μg/mL)	32	–	8	32	2	–
	Fold reduction			**8**		**16**

**Table:** 

		**AGL-726**							
		***A. baumannii*** **1605**				***A. baumannii*** **5075**			
**Antibiotics**		**0 μM**	**10 μM**	**15 μM**	**30 μM**	**0 μM**	**10 μM**	**15 μM**	**30 μM**
Imipenem	MIC (μg/mL)	32	16	8	–	32	–	8	4
	Fold reduction		**2**	**4**				**4**	**8**
Meropenem	MIC (μg/mL)	32	8	6	–	32	–	4	2
	Fold reduction		**4**	**5.3**				**8**	**16**
Doripenem	MIC (μg/mL)	32	8	6.0	–	32	–	6	1
	Fold reduction		**4**	**5.3**				**5.3**	**32**

**Table:** 

		**AGL-833**					
		***A. baumannii*** **1605**			***A. baumannii*** **5075**		
**Antibiotics**		**0 μM**	**2 μM**	**4 μM**	**0 μM**	**2 μM**	**4 μM**
Imipenem	MIC (μg/mL)	32	32	4	32	16	8
	Fold reduction		**0**	**8**		**2**	**4**
Meropenem	MIC (μg/mL)	32	32	4	32	32	4
	Fold reduction		**0**	**8**		**0**	**8**
Doripenem	MIC (μg/mL)	32	32	4	32	16	4
	Fold reduction		**0**	**8**		**2**	**8**

### Interactions between 2-AIs and response regulators

To further understand how 2-AIs are interacting with response regulators, we turned to structural biology techniques. The highly flexible nature of response regulators makes solving the full-length structure difficult. To date, we have solved the structures of the N-terminal receiver domains of QseB and BfmR using x-ray crystallography [PDB ID 5UIC and 5HM6 (Milton et al., 2017; Draughn and Milton et al. unpublished)], as well as the C-terminal DNA binding domain of BfmR [PDB ID 2NAZ (Draughn and Milton et al. unpublished)]. These structural domains can be combined using chemical crosslinking and molecular dynamics simulations to model full length response regulators (Olson et al., 2013, Draughn and Milton et al. unpublished). We have further employed molecular dynamics simulations and docking procedures to shed light on the interactions between 2-AIs and response regulators.

In lieu of a complete structure of QseB, we have used a homology model of PmrA from *Klebsiella pneumoniae* [PBD ID 4S04 and 4S05 (Lou et al., 2015)]. QseB and PmrA share 43% sequence identity and 61% sequence homology (Supplementary Figure 3). Alignment of the QseB N-terminal domain [PDB ID 5UIC (Milton et al., 2017)] with PmrA [PDB ID 4S05 (Lou et al., 2015)] crystal structures results in a Cα RMSD of 1.805 Å. Full length structures indicate that PmrA has a linker length of ~6 amino acids. Based on sequence alignment, we predicted that QseB has an ~8 amino acid linker. Combined, this information suggests that our PmrA derived model is a suitable stand-in for full length QseB. This model has allowed us to probe potential interactions between QseB and 2-AIs (Milton et al., 2017). From these studies, a binding interface between the N- and C-terminal domains was identified as the highest potential binding site. Similarly, an early model of BfmR identified the same 2-AI binding site (Thompson et al., 2012). This observation was supported by experimental finds which demonstrated that the N- and C-terminal domains of BfmR could bind a 2-AI independently. Both N—and C—terminal domain constructs of BfmR were independently pulled down by a 2-AI compound (Thompson et al., 2012). This suggests that a compound binding site lies at the interface between the two domains. All subsequent docking experiments for BfmR and QseB have identified some variation of the N- and C-terminal domain interface as the most favorable binding site for 2-AI compounds. Further structural studies will be necessary to confirm that the domain interface is the binding site and elucidate the specific residues that facilitate 2-AI compound binding.

As a follow up to prior studies employing docking with the current generation of 2-AI compounds (Milton et al., 2017), we performed “large-box” blind docking using Autodock VINA to the solution equilibrated “tucked” state of QseB. The low MMGBSA scored pose positions for compounds lie in cavities at the interface between the N- and C-terminal domains (Supplementary Figure 4). These poses have low RMSD excursions over 30–60 ns while bound to the response regulator. This suggests that the compound binding sites identified are temporally stable and have reasonable residence times for ligands that bind with μM affinity.

**Figure 4:**
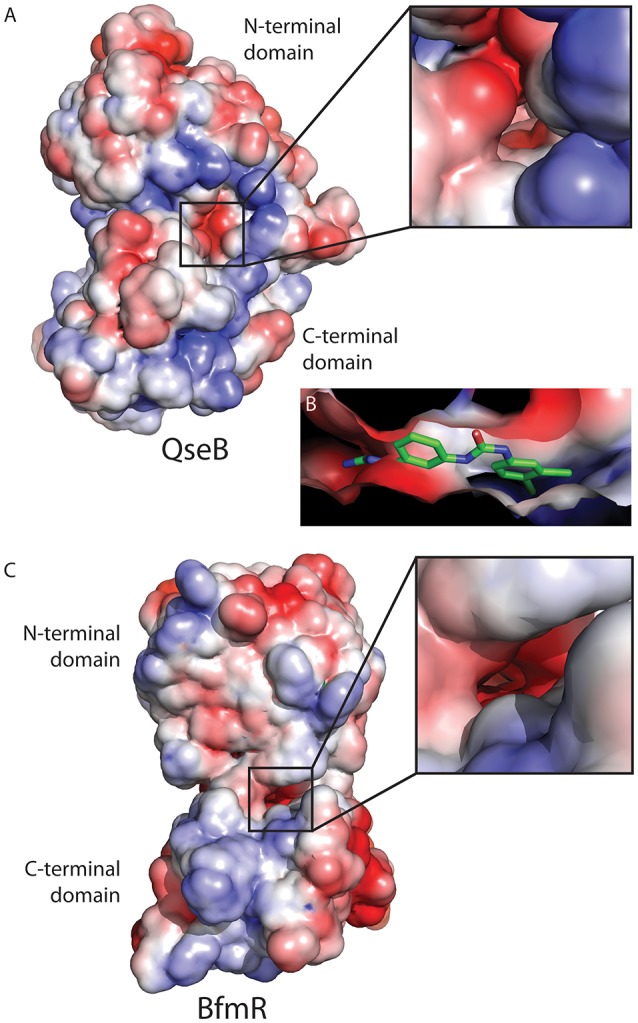
Electrostatic map of QseB and BfmR. **(A)** QseB has an electronegative interface between the N- and C-terminal domains. Negative charges extend deep into the protein as shown in the blow out. **(B)** Docking of AGL-726 places the compound into this interface. The 2-aminoimidazole head group lines up with the electronegative region, suggesting the head group neutralizes the negative charges. **(C)** BfmR also contains a deep electronegative interface created between the N- and C-terminal domains. Electrostatic potential ranges from −2 (red) to +2 (blue), with 0 shown in white.

Examining the electrostatic properties of the interface between the N-terminal and C-terminal domains reveals an electronegative region in both QseB and BfmR. A concentrated electronegative region lines the inside of the interface central to the protein. This region is adjacent to where the flexible linker connects the two domains (Figures 4A,C). Similar patterns were observed in other full length response regulators ComE [PDB ID 4CBV (Boudes et al., 2014)] and KdpE [PDB ID 4KNY and 4KFC (Narayanan et al., 2014)], described below. Oddly, PmrA, for which the model of full length QseB is based, does not appear to have an electronegative interface [PDB ID 4S04 and 4S05 (Lou et al., 2015)]. This suggests that full length structural information will be very important for designing potent inhibitors. Docking of AGL-726 to QseB positions the compound within this interface (Figure 4B and Milton et al., 2017). The 2-aminoimidazole head group docks within the electronegative region. This positioning was observed with many other 2-AIs (data not shown). BfmR and QseB share similar electrostatic topologies, providing evidence as to why the 2-aminoimidazole head group is required for compound efficacy. Depending on the response regulator, we propose that compounds can be tailored to interact with the exterior region of the interface while the conserved head group binds the electrostatic residues on the interior. This observation will help to guide the next generation of 2-AI derivatives.

### Response regulator dynamics

Flexibility likely plays a critical role in a response regulator's ability to bind a variety of target DNA sequences. In fact, a DNA substrate is often used to lock down the mobile C-terminal domains in crystal structures. In order to understand how our compounds bind response regulators, it is important to understand the dynamics of the system.

**Figure 5:**
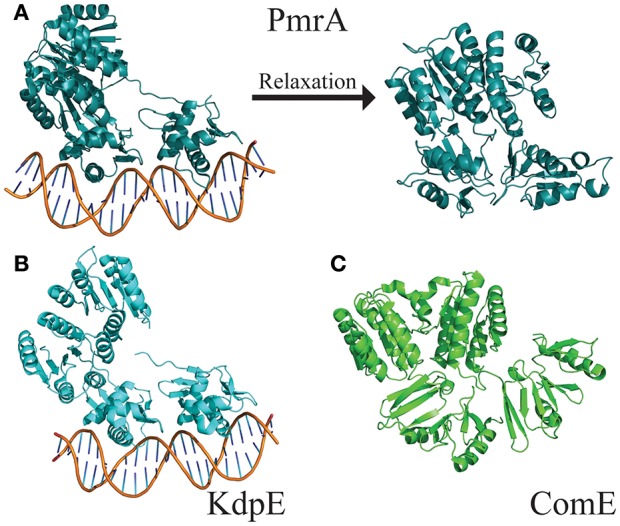
Structures of full length response regulators show two conformations. **(A)** PmrA (PDB ID 4S05) is in a “tucked/extended” state when bound to DNA in a crystal. Molecular dynamics of solution state PmrA in the absence of DNA relaxes to a “tucked/tucked” state. Structure model represents the top scoring pose from each cluster based on free energy minimization. **(B)** Crystal structure of KdpE bound to DNA (PDB ID 4KNY) also is in a “tucked/extended” conformation. **(C)** The apo structure of ComE (PDB ID 4CBV) is in a “tucked/tucked” state similar to the relaxed PmrA.

The flexible linker connecting the N- and C-terminal domains allows response regulators to sample a wide range of states. The “tucked” and “extended” states describe the relationship of the C-terminal DNA-binding domain to the N-terminal dimerization domain (Milton et al., 2017). These two extreme states have been observed in the crystal structures of PmrA from *K. pneumoniae* [PBD ID 4S04 and 4S05 (Lou et al., 2015)] and KdpE from *Escherichia coli* [PDB ID 4KFC and 4KNY (Narayanan et al., 2014)] (Figures 5A,B). Both structures are of DNA bound proteins that belong to the OmpR/PhoB family of response regulators. KdpE has a linker length of ~8 amino acids and shares a 31% identity with 56% homology to QseB and 33% identity with 55% homology to PmrA (Supplementary Figure 3). Secondary structures of the N-terminal and C-terminal domains of QseB, PmrA, and KdpE are nearly identical structures with Cα RMSDs of ~1.1 Å for the N-terminus and ~1.5 Å for the C-terminus. Based on these crystal structures, thermal shift binding data, and molecular docking simulations, we previously proposed that compounds could bind either the “tucked” or “extended” state. In the model, compounds that favored the “tucked” conformation were tighter binders and more potent inhibitors. Molecular dynamics simulations allow for the model to be further explored.

**Figure 6:**
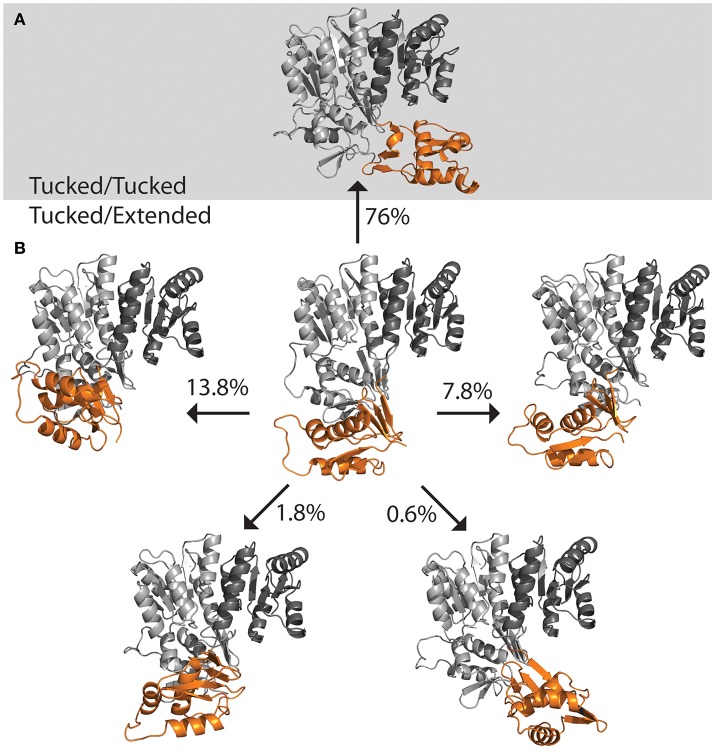
Population distribution of PmrA. Having started with an “extended” state, the populations shifts to predominately “tucked” on a short time scale. **(A)** Molecular dynamics of PmrA (PDB ID 4S05) quickly relaxes to a “tucked/tucked” state and samples this state 76% of the time at a 250 ns time scale. **(B)** The PmrA crystal structure bound to DNA (center) begins in a “tucked/extended” state. Variations on the “tucked/extended” state are sampled for very short periods of time and account for small portions of the population at a given time. Structure models represent the top scoring pose from each cluster based on free energy minimization.

The DNA was first removed from the full length crystal structure of PmrA [PBD ID 4S05 (Lou et al., 2015)] and immersed in a large box of TIPS3P water. Molecular dynamics studies revealed that, following early equilibration, the “extended” chain of PmrA rapidly collapsed to a “tucked” conformation (Figure 5A) within the first 70 ns of the 250 ns simulation at 300 K. This suggests that there is likely a small or no energy barrier to folding to the “tucked” state. Examination of trajectory movies suggests the timescale for compaction may possibly be determined by the exclusion of intervening water molecules between the two C-terminal domains and low-energy gating transitions of a few residues in the linker chain. Following the C-terminal conformational transition, PmrA remains “tucked” with smaller scale dynamical fluctuations (Figure 6). Examining clustered conformations for the full duration of the simulation with this large amplitude transition, approximately 76% of the sampled populations take on a “tucked” conformation in the absence of DNA, with variations of the “extended” state present at short times. These percentages are not steady-state populations, *per se*, but merely reflect the conformational preferences for a simulation of this length. Supplementary Figure 5 shows that between 50 and 70 ns there is a significant increase in the RMSD of the sampled conformations as compared to the original starting structure. Investigation of the Cα fluctuations at 70 ns reveals that the major contributor to change in RMSD is due to significant movement of the C-terminal residues of the “extended” state chain (Supplementary Figure 6). This is what one would expect for relaxing a system to a compact/energetically stable regime from a higher energetic extended state. Interestingly, the full length crystal structure of ComE [PDB ID 4CBV (Boudes et al., 2014)] has both dimer chains in the “tucked” conformation (Figure 5C). The lack of DNA may be attributed to this conformation. ComE is a member of the AlgR/AgrA/LytR family of transcription regulators. Its C-terminal DNA binding domain is distinct from the winged-helix-turn-helix found in OmpR/PhoB family. ComE does share some structural similarities to QseB, PmrA, and KdpE. A ~10 amino acid linker connects the two domains of ComE. The N-terminal domain has a 25% identity and 46% homology with C α RMSD of 3.848 Å to QseB (Supplementary Figure 3). Since no full length structures of OmpR/PhoB response regulators in a dimer conformation have been solved, ComE is the closest representation of a non-DNA bound response regulator dimer. Monomeric OmpR/PhoB family structures such as DrrB and MtrA have been solved in a “tucked” conformation, further suggesting that the “tucked” state is more favorable in solution, in the absence of DNA.

**Figure 7:**
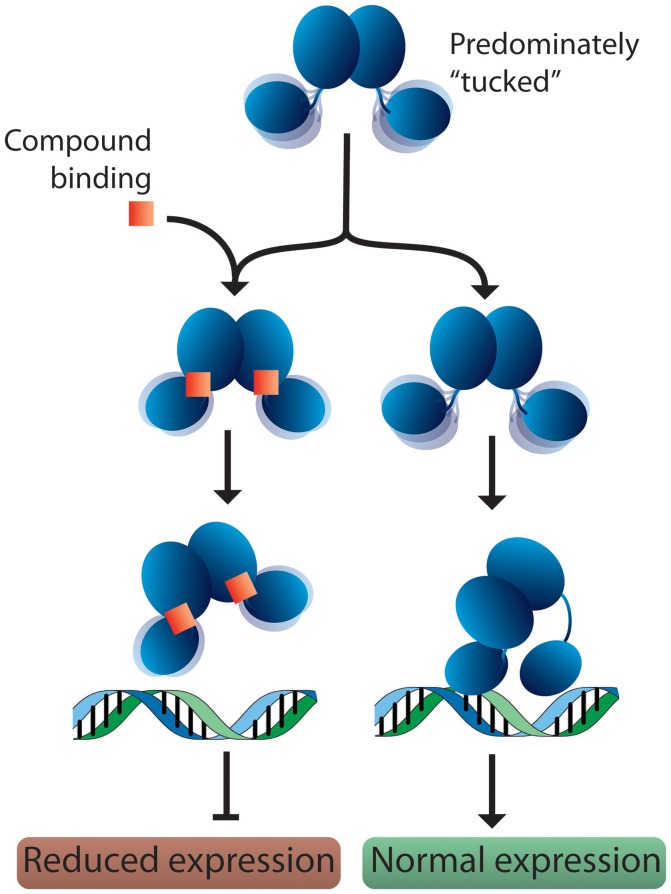
Proposed model for response regulator DNA binding and 2-AI binding. In solution, response regulators are predominantly in the “tucked” conformation. In the absence of inhibitor, the response regulator is free to move the C-terminal domains. This assists in binding the target DNA and allows the N-terminal domain to “kneel” over one of the DNA-binding domains. Binding of a 2-AI compound likely increases the interactions between the N- and C-terminal domains, impacting the protein's ability to position both DNA-binding domains on the promotor substrate and “kneel” over the N-terminal domain. Interfering with DNA binding or the protein conformation on the DNA would be expected to reduce the expression of downstream targets.

Based on these observations, we propose a potential mechanism for response regulator DNA binding. In solution, response regulators preferentially adopt a tucked conformation, occasionally sampling more extended poses. When a DNA substrate is identified, the N-terminal domain “kneels” over one of the C-terminal domains (Figure 7). This movement results in one dimer chain becoming tucked while the linker region of the other chain stretches out. Since 2-AIs likely bind to the interface between the N- and C-terminal domains, our working model proposes that they may impact the ability of the response regulator to “kneel” upon DNA binding (Figure 7). Both DNA binding domains are likely needed to sufficiently bind the target DNA sequence. Thus, a shift in equilibrium between “tucked” and “extended” states or trapping the response regulator in a “tucked” conformation may impact DNA binding and/or regulation of downstream targets. Further studies are needed to validate this hypothesis. With this in mind, 2-AIs designed to tightly bind the interface and facilitate interactions with both the N- and C-terminal domains should be excellent inhibitors.

## Conclusion

Using a combination of biochemical and cellular techniques, we confirmed that BfmR is a cellular target of 2-AI compounds. These results agree with our similar findings for QseB. Sampling of a library of compounds allowed for comparisons of behavior across multiple techniques, from which information about the most promising compounds can be deduced. Based on these experiments, AGL-726 and AGL-833 appear to have significant therapeutic potential, due to their ability to bind BfmR with relatively high affinities, inhibit biofilm formation, and increase sensitivity to carbapenem derivatives. The confirmation of both QseB and BfmR as targets of 2-AI compounds and the identification of the same lead compounds suggests that studies to determine the inhibition mechanism in one system will translate to the other. Since *Francisella* encode only three response regulators, *Francisella* could be a model system for the determination of a 2-AI mechanism of action in *A. baumannii*. Understanding the interactions between response regulators and 2-AIs on a structural level is necessary to fully understand the mechanism of inhibition. This information will play a vital role in the development of even more potent compounds to combat antimicrobial resistance. Molecular dynamics simulations suggest that response regulators are prone to spend a majority of their time in the “tucked” state. As such, this state should be targeted for future drug design. We hypothesize that the binding of a 2-AI into the interface between the N—terminal and C—terminal domains will increase the interaction between these domains, stabilizing the “tucked” state. This binding could result in reduced sampling of the “extended” state. The work presented here lays the ground work for understanding how 2-AI compounds inhibit response regulators. Further studies are necessary to validate this working model. A better understanding of how 2-AIs interact with response regulators and the mechanisms involved in DNA binding will inform the development of more potent libraries of compounds with specific and broad range targets.

## Author contributions

MM: designed the research, performed biochemical experiments and wrote the manuscript; MM and CL: expressed and purified proteins; MM, BM, and BK: performed cellular experiments; DH: performed molecular dynamics studies; DJ: synthesized all compounds; DZ: provided all synthesized compounds; RT, RM, CM, and JC: supervised research.

### Conflict of interest statement

Authors BK, DJ, and DZ were employed by the company Agile Sciences, Inc. The other authors declare that the research was conducted in the absence of any commercial or financial relationships that could be construed as a potential conflict of interest.
