# Supplementary material for: Re-sensitizing Multidrug Resistant Bacteria to Antibiotics by Targeting Bacterial Response Regulators: Characterization and Comparison of Interactions between 2-Aminoimidazoles and the Response Regulators BfmR from Acinetobacter baumannii and QseB from Francisella spp
Source: Front Mol Biosci. 2018 Feb 13;5:15. doi: 10.3389/fmolb.2018.00015 (PMC5816815; doi:10.3389/fmolb.2018.00015)
Supplement: Supplementary file 1 [file DataSheet1.pdf]

Supplementary Material

Re-sensitizing Multidrug Resistant Bacteria to Antibiotics by Targeting Bacterial Response Regulators: Characterization and Comparison of Interactions Between 2-Aminoimidazoles and the Response Regulators BfmR from *Acinetobacter baumannii* and QseB from *Francisella* spp.

Morgan E. Milton, Bradley M. Minrovic, Danni L. Harris, Brian Kang, David Jung, Caleb P. Lewis, Richele J. Thompson, Roberta J. Melander, Daina Zeng, Christian Melander, and John Cavanagh

1 Supplementary Figures and Tables

1.1 Supplementary Figures

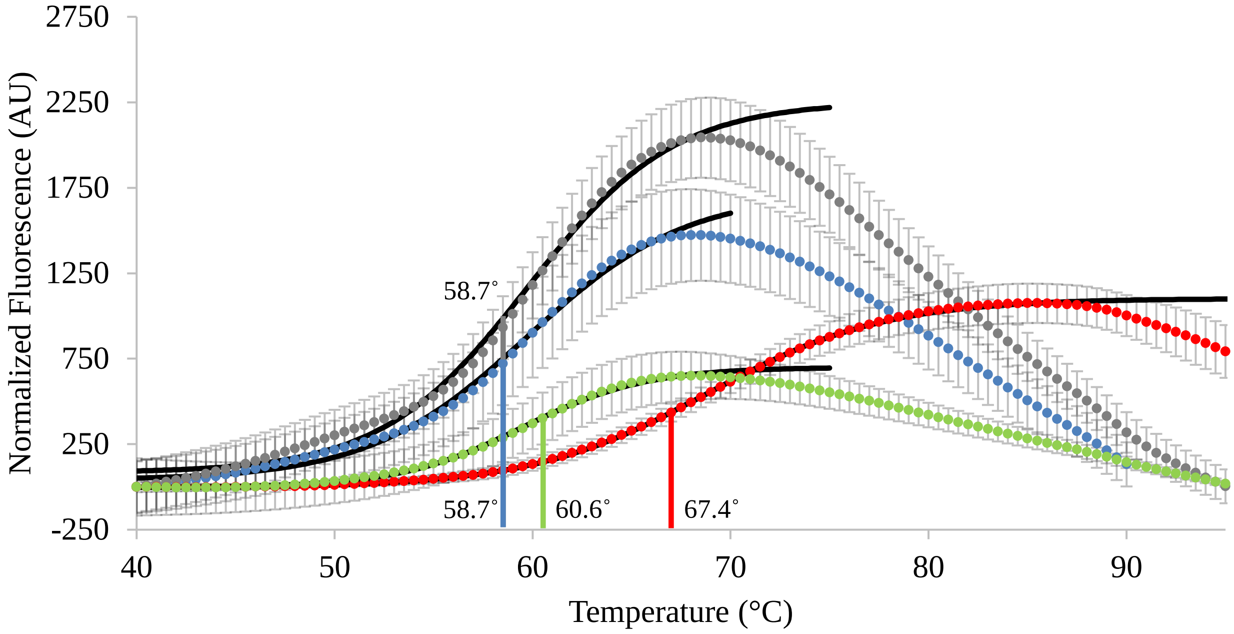

Supplementary Figure 1. Representative raw data of thermal shift profiles for apo-BfmR (blue), BfmR with AGL-600 (grey), BfmR with AGL-726 (red), and BfmR with AGL-833 (green). Error bars represent the standard deviation in fluorescence intensity from at least three technical replicates. Raw data is fit to a sigmoidal Boltzmann curve using SigmaPlot (black lines). The inflection point (marked with vertical lines) of the fit curve denotes the T_m of the protein. The T_m for each condition is labeled. T_m values from three experiments were then averaged to produce the final temperatures.

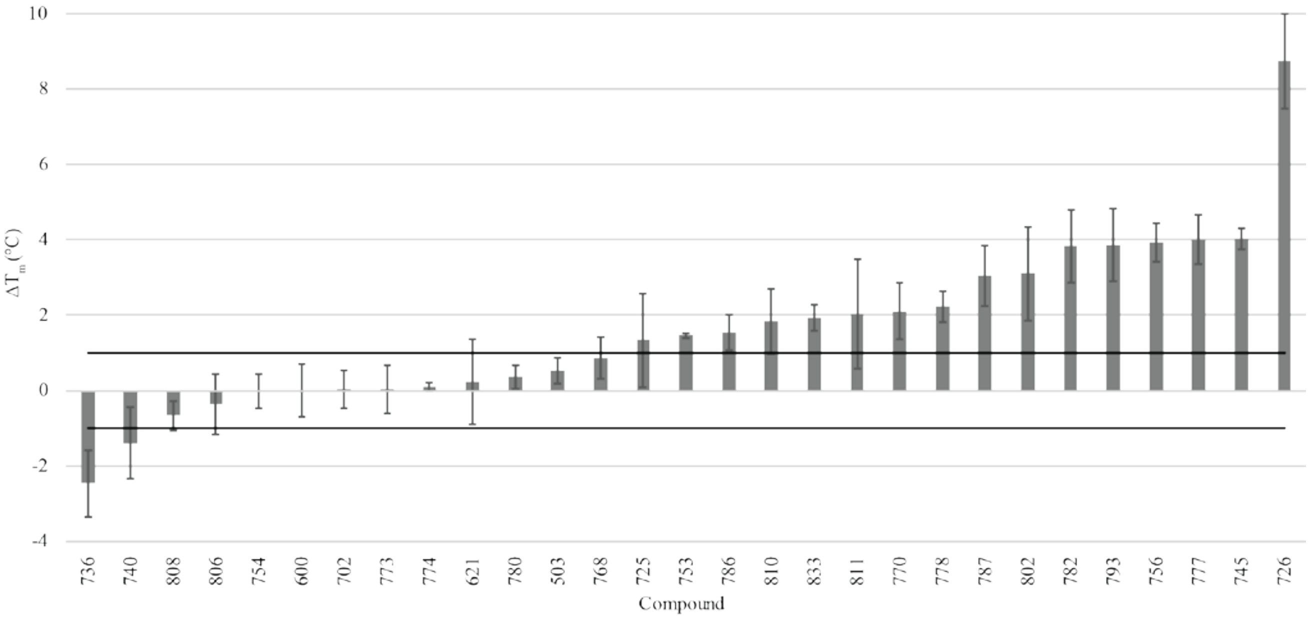

Supplementary Figure 2. Thermal shift of BfmR upon addition of 2-AI compounds. The average change in melting temperature is plotted for each 2-AI compound tested. The thick horizontal black lines represent the standard deviation of the average melting temperature of BfmR in the absence of any compound. The compound identification number is listed on the x-axis. Assay was performed in triplicate.

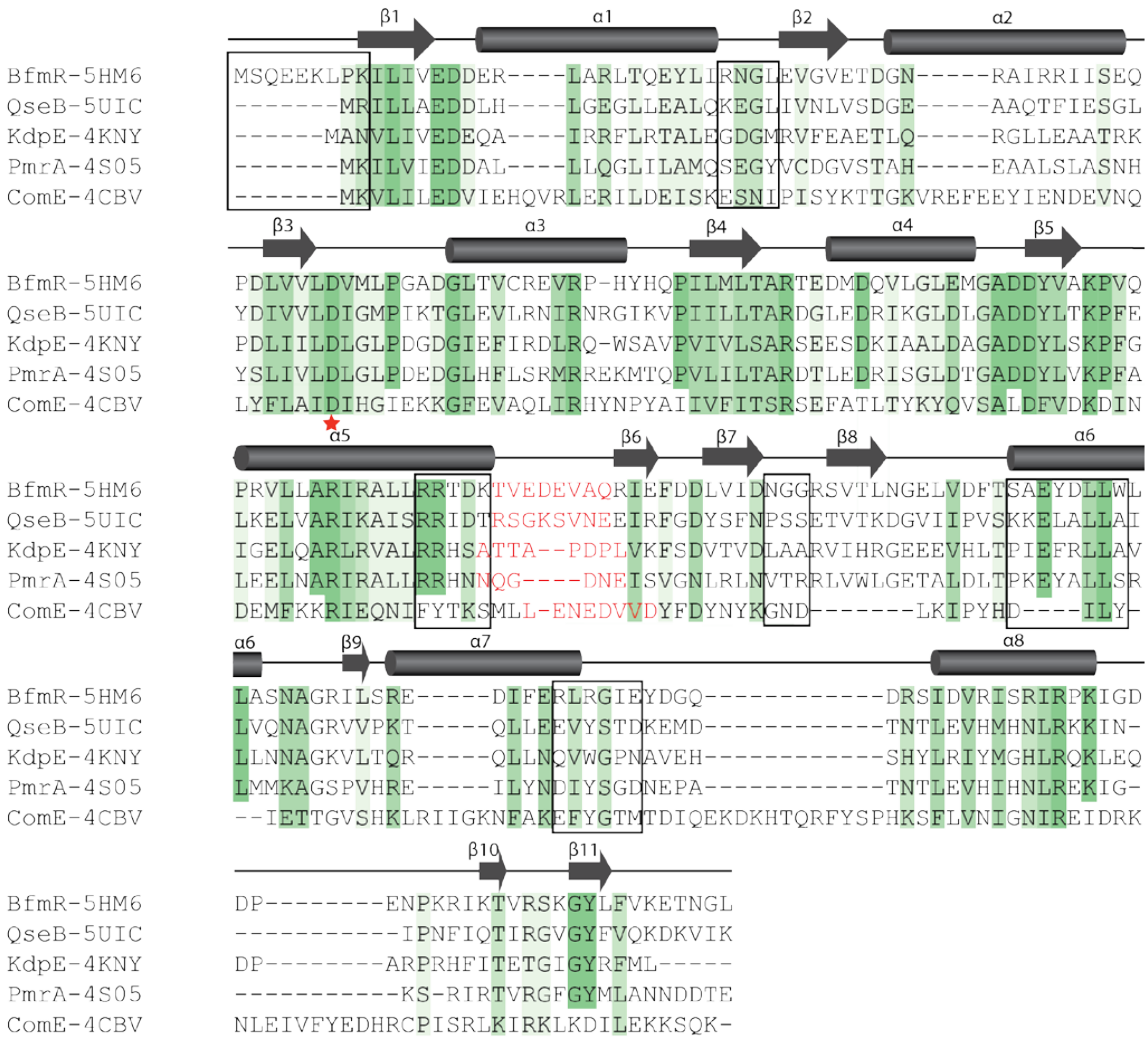

Supplementary Figure 3 Sequence alignment of response regulators. Approximate secondary of OmpR/PhoB family response regulators (BfmR, QseB, PmrA, and KdpE) is represented by arrows for sheets and cylinders for helices. Conserved residues cluster around the phosphorylation site (marked with a red star). Linker regions are highlighted in red. Boxed regions are the residues predicted to compose the “tucked” binding pocket. These residues may facilitate the binding of 2-AI compounds. Alignment generated using Clustal Omega.

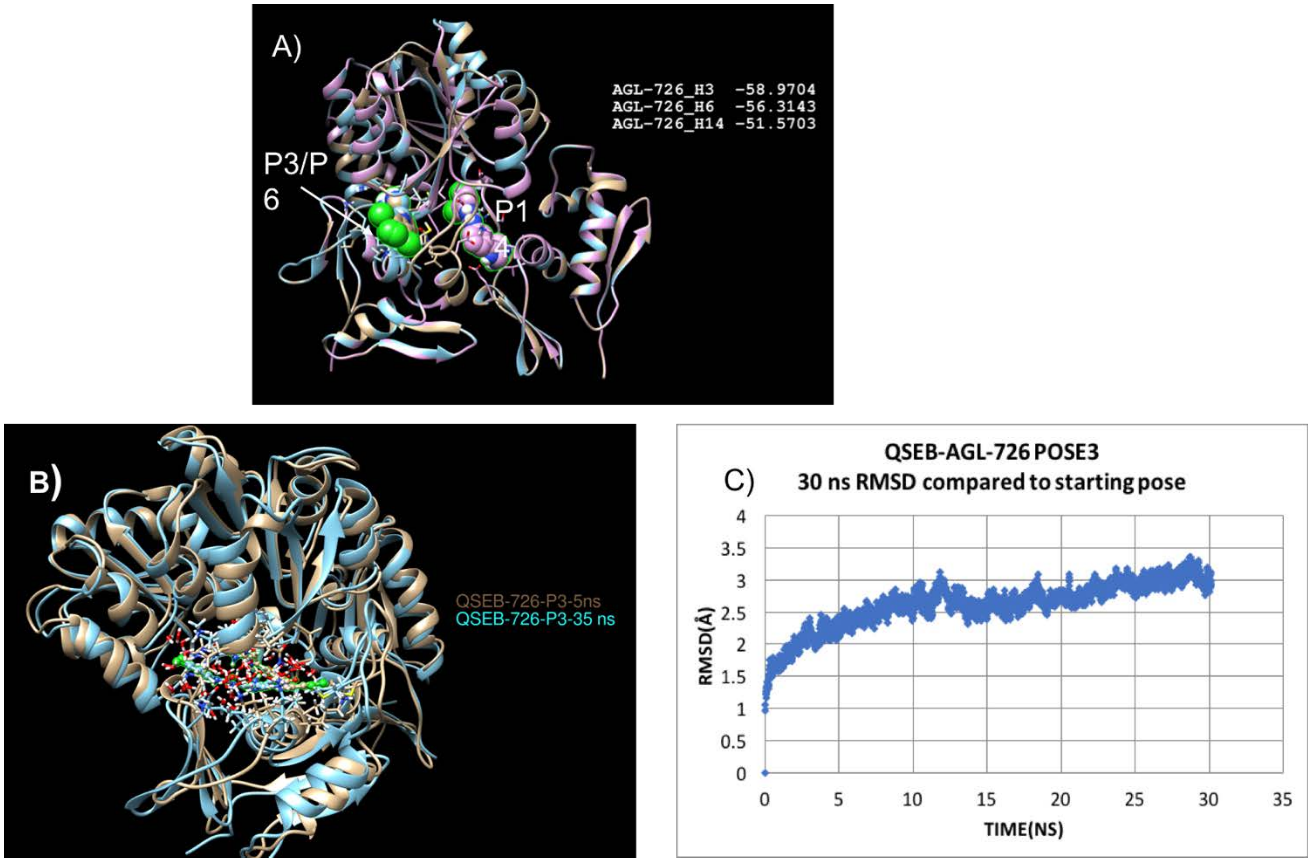

Supplementary Figure 4 Docking of AGL-726 to QseB. (A) The lowest three MMGBSA-Min rescored Autodock VINA poses of AGL-726 in the relaxed, solution equilibrated, “tucked” conformation of QseB. The lowest energy poses lie at the interface between the N- and C-terminal domains of QseB and have binding energies of -58.9704, -56.3143, and -51.5703 kcal. (B) and (C) show that while some structural rearrangement of AGL-726 occurs in the binding pocket over ~30 ns, the deformation is relatively small, where the interior portion of the ligand is virtually immobile with higher mobility for the portion of the ligand near the solvent interface. The inhibitor remains in the binding site cleft between the N- and C-terminal domains.

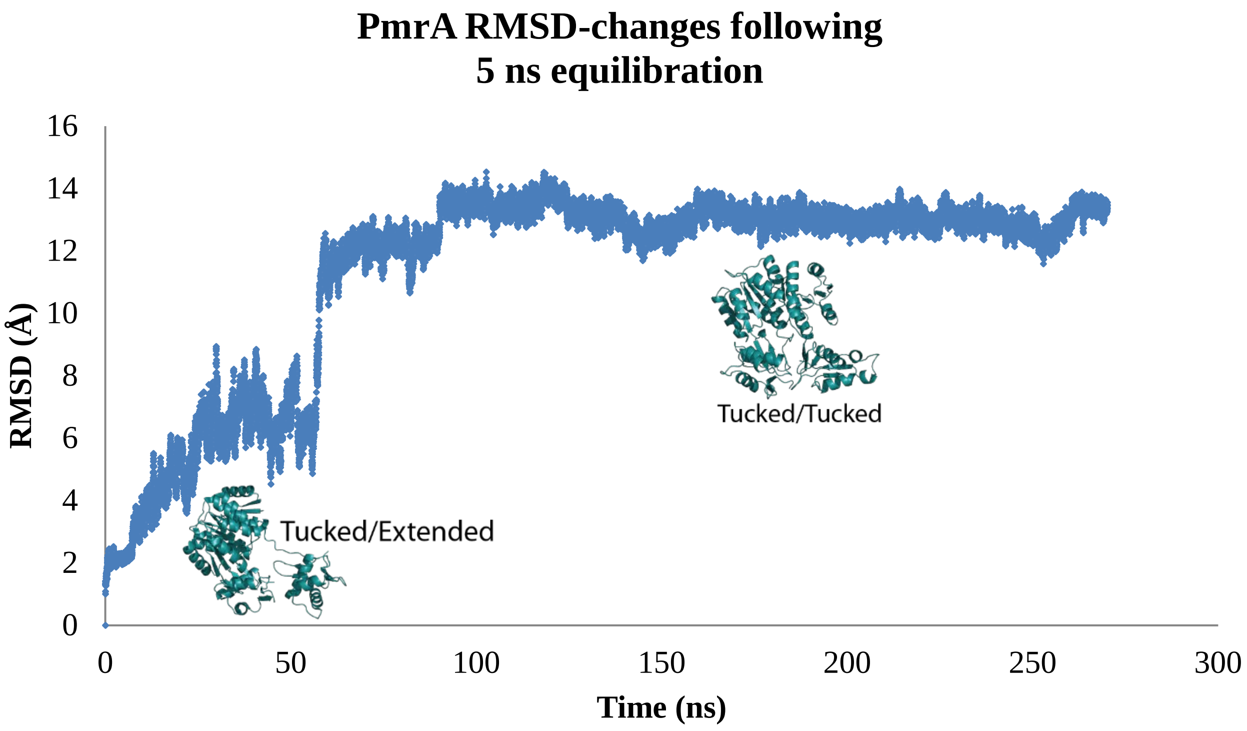

Supplementary Figure 5 RMSD of PmrA (PDB ID 4S05) molecular dynamics simulations in the absence of DNA as compared to the original starting structure. Dynamics were performed as an unconstrained aqueous simulation over 250 ns. C-terminal relaxation from an "extended" to "tucked" state occurs during the early 70 ns time scale and is responsible for the RMSD-time-series appearance shown here.

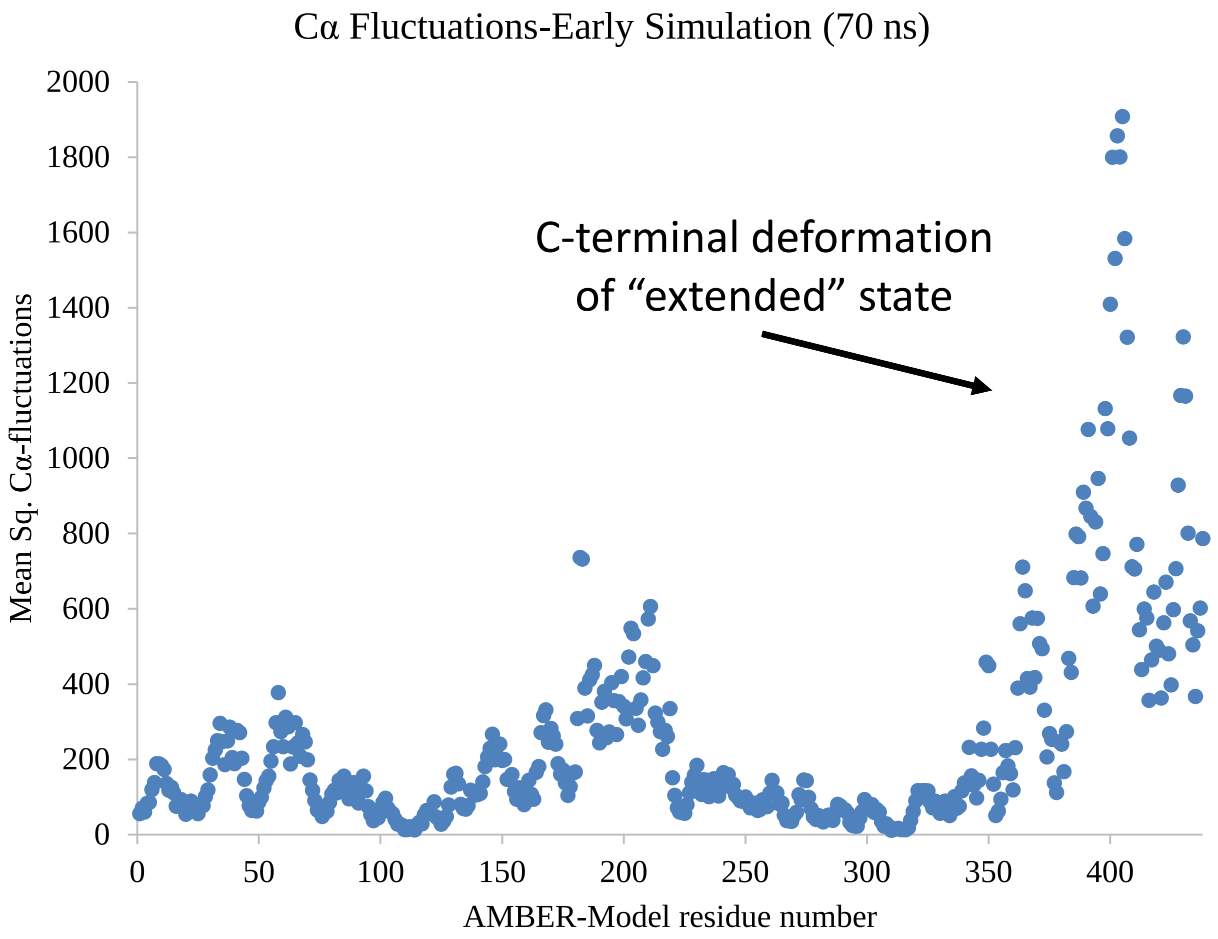

Supplementary Figure 6 Evaluation of the fluctuation of the Cα positions during early (70 ns) aqueous dynamics simulations reveals that a majority of the changes occur in the C-terminal domain of the “extended” state chain. The C-terminal domain transition predominately takes place between 50 and 70 ns.

1.2 Supplementary Tables

Supplementary Table 1. MIC lowering activity for 2-AI library

		A. baumannii 1605			*A. baumannii* 5075		
		imipenem	meropenem	doripenem	imipenem	meropenem	doripenem
	Compound (uM)	MIC (mg/mL)	MIC (mg/mL)	MIC (mg/mL)	MIC (mg/mL)	MIC (mg/mL)	MIC (mg/mL)
no compound	-	32	32	32	32	32	32
503	60	4					
600	60	4	4	8			
	30				2	2	2
621	60		8		8	4	
	30	4	16	6		16	2
702	60		32		32	32	32
725	60	2	2	1	4	1	0.5
	30				4	2	2
	15	8	8	8	8	8	8
	10	16	16	8			
726	30				4	2	1
	15	8	6	6	8	4	6
	10	16	8	8			
736	60				32	32	32
740	60				32	32	32
745	60	4	4	2	4	4	2
	30	4	4	4	4	4	4
	15				4	4	4
	10	8	8	8	8	8	8
753	60				4	4	4
	30		16		8	8	8
754	60	4	2	2	2	1	1
	30				8	8	4
	15				32	32	32
756	15	3	2	2	4	4	2
	10	4	4	8	16	12	6
	5	16	16	16			
768	60				1	0.5	0.5
	30	1	2	1	2	2	1
	15	4	4	4	16	6	8
	5	32	32	32			
770	15				4	4	8
	10	4	4	8	8	4	8
	7	16	16	8			
773	60	2	2	2	64	64	128
	30	3	2	4			
	5	16	16	32			
774	60				4	2	1
	30	16	8	8	8	8	4
777	60				16	32	32
778	30	2	2		2	1	1
	15	8	8	4	4	2	2
	10				4	4	4
	5				16	16	8
780	60				32	32	32

782	60<br>30		8		8	8	8
786	30<br>15<br>5	6	30<br>4	4	3<br>8<br>16	3<br>4<br>32	2<br>4<br>16
787	60<br>30<br>15<br>10	1<br>8<br>16	2<br>8<br>16	2<br>6<br>16	3<br>4<br>8<br>8	2<br>2<br>4<br>8	2<br>3<br>4<br>8
793	7<br>5	3<br>8	6<br>16	4<br>16	12<br>16	8<br>12	6<br>16
802	10<br>7<br>5	4<br>16	4<br>16	4<br>16	4<br>32	4<br>16	4<br>16
806	60	32	32	32	32	32	32
808	60	32	32	32	32	32	32
810	60	32	32	32	32	32	32
811	4	16	32	32	32	32	16
833	4<br>2	4<br>32	4<br>32	4<br>32	8<br>16	4<br>32	4<br>16
